# Data Refinement and Channel Selection for a Portable E-Nose System by the Use of Feature Feedback

**DOI:** 10.3390/s101110387

**Published:** 2010-11-17

**Authors:** Sang-Il Choi, Su-Hyun Kim, Yoonseok Yang, Gu-Min Jeong

**Affiliations:** 1 School of Electrical Engineering and Computer Science, Seoul National University 1, #047, San 56-1, Sillim-dong, Gwanak-gu, Seoul 151-744, Korea; E-Mail: kara@csl.snu.ac.kr; 2 Electrical Engineering, Kookmin University 2, 861-1, Jeongeung-dong, Songbuk-gu, Seoul 136-702, Korea; E-Mail: matery@naver.com; 3 Biomedical Engineering, Chonbuk National University 3, 664-14, Iga Deokjin-dong, Jeonju, Korea; E-Mail: ysyang@chonbuk.ac.kr

**Keywords:** e-nose system, vapor classification, feature feedback, discriminant feature

## Abstract

We propose a data refinement and channel selection method for vapor classification in a portable e-nose system. For the robust e-nose system in a real environment, we propose to reduce the noise in the data measured by sensor arrays and distinguish the important part in the data by the use of feature feedback. Experimental results on different volatile organic compounds data show that the proposed data refinement method gives good clustering for different classes and improves the classification performance. Also, we design a new sensor array that consists only of the useful channels. For this purpose, each channel is evaluated by measuring its discriminative power based on the feature mask used in the data refinement. Through the experimental results, we show that the new sensor array improves both the classification rates and the efficiency in computation and data storage.

## 1. Introduction

An electronic nose (e-nose) is an instrument that is designed to detect and discriminate vapors using an array of sensors [1–6]. In an early electronic nose, calorimetric sensors were used to perform measurements on vapors, and the measurements were usually expressed in arrays of colors, *i.e.*, in the form of colored images [7]. Such an e-nose system, which was used only in a laboratory environment, utilized complicated analytic procedures including the use of precise equipment such as gas chromatography (GC) system or a mass spectrometer (MS) combined with sophisticated machine intelligence. With recent advances in electrochemical sensor and digital technologies, the e-nose system can now support a more portable and intelligent platform in the collection and processing of gas compounds. Compared to the laboratory analytic system, the portable e-nose has advantages in that it can simply and frequently measure odors in daily environments, and thus it can be applied to a variety of fields, which include environmental protection [8], the food industry [9], the detection of explosive substances [10] and medical diagnosis such as the identification of infections through the examination of odors in breath or tissues [11].

A portable e-nose system is composed of a sensor array that contains several channels and a classifier. Each channel, which is one element (individual sensor) of sensor array, has a different characteristics [7]. The sensors are made of polymer carbon composite materials for high sensitivity and selectivity. By use of the information acquired from those sensor arrays, the classifier distinguishes different vapors by a classification rule. In order to make an e-nose system perform reliably in various environments, it needs improvements not only in sensor hardware aspects, but also in data mining methods that process and classify the data measured by the sensors. In [7], the multi-channel acquisition of the sensor array responses in two-dimensional image form and high contrast discrimination is accomplished in different volatile organic compounds (VOC) by template matching. Some feature extraction or selection methods can be used effectively to classify vapors for the portable e-nose system. The measurement for a data sample over *n* time points is represented as a vector that consists of *n* input variables. In [12], the discriminant features for classification are extracted by LDA (Linear Discriminant Analysis) and one nearest neighbor classifier is used with the features. In [13], the hierarchical classification method that combines Fisher discriminant analysis and modified Sammon mapping was proposed. Some vector machines such as the support vector machine or relevance vector machine were used to classify vapors [14,15]. However, since a feature extraction method makes use of all the input variables of the original data to extract new features, if there are problems in the data collection process or if there are input variables that are corrupted by noise, the feature extraction results may be compromised and so are the final results. Therefore, in order to make the extracted features robust against noise, some input variables that contain a large amount of discriminative information should be distinguished before the extraction of discriminant features for classification.

In this paper, we propose a new data refinement and channel selection method for vapor classification. We develop the method based on feature feedback for e-nose data [16]. The feature feedback is conducted as follows: (1) extraction of features from the original data; (2) making a reverse mapping from the extracted feature space to the original input space; (3) extraction of discriminant features which are used as a classifier. By doing this, we can reduce the redundancy and noise in the data, and consequently can expect to improve classification performance.

In addition, we can design the e-nose system more effectively by selecting necessary channels while discarding unnecessary channels. Especially, in the case that the kinds of vapors to be recognized are restricted to particular gases, we can evaluate the contribution of each sensor to the classification based on the results of feature feedback.

By selecting and using only the important sensors which contribute to classification, we can reduce the size of the sensor array while preserving or improving the classification performance. It is an important consideration to minimize the data and computation in portable systems due to the limitation of the available processor and operation power. Through the experimental results, we show that the proposed method can improve not only the classification rates but also the efficiency by redesign of the sensor array.

The rest of this paper is organized as follows. In Section 2, we briefly overview the related works for discriminant feature extraction and feature feedback. We present how to refine the originally measured data by removal of the unimportant part and also show how to select useful channels in Sections 3 and 4, respectively. In Section 5, we describe the experimental results and the conclusion follows in Section 6.

*W^L^* of LDA can be computed from the eigenvectors of $S_{W}^{-1}S_{B}$ corresponding to the *n*′ largest generalized eigenvalues. In Table 1, the columns of $W^{F}=[w_{1}^{F}w_{2}^{F}..w_{n\prime }^{F}]$, *F* ∈ {*P*, *L*}, are the projection vectors. In the *n*′-dimensional feature space, the sample **x***_k_* is represented as a low-dimensional feature vector **y***_k_* = (*W^F^*)*^T^* **x***_k_*, *F* ∈ {*P*, *L*}.

## 2. Related Works

### 2.1. Feature Extraction

There are a number of widely known feature extraction methods such as PCA (Principal Component Analysis) [17] and LDA (Linear Discriminant Analysis) [18], and further improvements to these methods are still being made [19,20].

Let us consider a set of *N* data samples, each of which can be a point **x***_k_* ∈ ℝ*^n^* in an *n*-dimensional vector space. PCA finds the best set of projection vectors in the sample space that maximizes the total scatter across all samples. LDA finds a set of projection vectors that maximizes the between-class scatter matrix (*S_B_*) while minimizing the within-class scatter matrix (*S_W_*), simultaneously. The scatter matrices and objective functions for PCA and LDA are shown in Table 1.

### 2.2. Feature Feedback

We first extract projection vectors that map the input space into the feature space by using a feature extraction method, and then feed the extracted features back to the input space. Based on the feedback information, each data sample is differentiated into two parts: the important and unimportant parts. For a data sample **x***_k_* that contains *n* input variables {*x_ki_*|*i* = 1,.., *n*}, let **e***_i_* ∈ ℝ*^n^* be the *i*-th unit coordinate vector of the input space and let **w***_l_* ∈ ℝ*^n^* be the projection vector corresponding to the *l*-th largest eigenvalue obtained by a feature extraction method introduced in the previous subsection. Then, **w***_l_* can be expressed by a linear combination of **e***_i_*s as follows:

$$w_{l}={\left[w_{l1},w_{l2},..,w_{ln}\right]}^{T}=w_{l1}e_{1}+w_{l2}e_{2}+..+w_{\ln}e_{n}$$

Here, the magnitude of *w_li_* indicates how much the *i*-th coordinate vector **e***_i_* contributes to the projection vector **w***_l_*. Therefore, if *w_li_* is larger in magnitude than *w_lj_* for a projection vector **w***_l_*, the coordinate vector **e***_i_* (*i.e.*, the *i*-th input variable) can be regarded as more important than the **e***_j_* (*i.e.*, the *j*-th input variable). Among the *n* variables in a data sample, we select the *t*(*< n*) variables corresponding to some largest values of |*w_li_*| in the order from greatest to least. Then the input variables corresponding to selected *t* variables are used for classification.

## 3. Data Refining Method to Improve Classification Performance

We develop the feature feedback method for vapor classification and apply it to refine the data acquired by the sensor arrays. The proposed data refining process is performed by the following two stages. At the first stage, the features are extracted from the original *N* data samples {**x**_1_,.., **x***_N_*}, **x***_k_* ∈ ℝ*_n_*, by the use of PCA, and then the feature feedback is applied to reduce the noise in the data. At the second stage, LDA is used to extract features from the resultant data of the first stage. We feed these features back to the data, and finally obtain the refined data by selecting the important part for vapor classification.

### 3.1. Noise Reduction by the Use of PCA Feature Feedback

**Figure 1.**
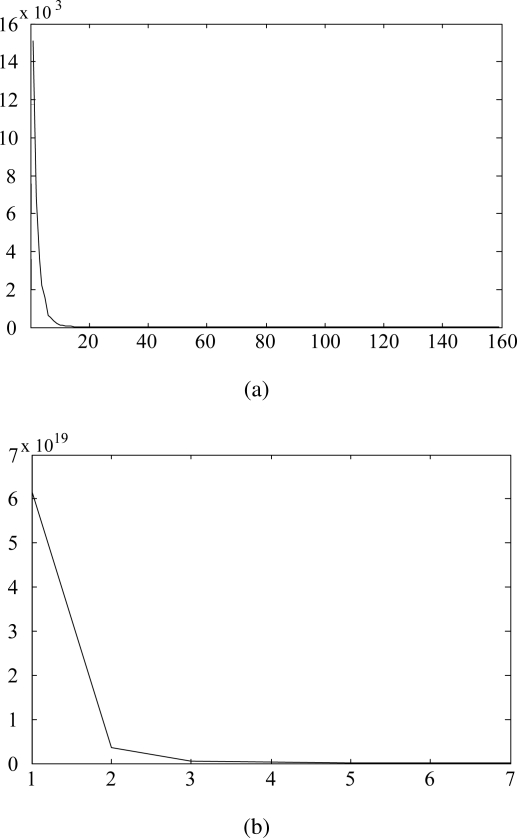
Eigenvalues in descending order. **(a)** PCA ( $\lambda_{l}^{P}$); **(b)** LDA ( $\lambda_{l}^{L}$).

In this paper, we utilize the VOC measurement data set used in the previous study: acetone, benzene, cyclohexane, ethanol, heptane, methanol, propanol, toluene [7]. The data set contains 160 samples and each sample consists of 32, 000 variables that were measured through 16 channels over 2,000 time points, *i.e.*, *N* = 160, *n* = 32,000. PCA finds a transformation by performing the eigenvalue decomposition of the total scatter matrix. Since small eigenvalues are very sensitive to noise, we can reduce the effect of noise by discarding the projection vectors with small eigenvalues. Figure 1(a) shows an example of eigenvalues $\left\{\lambda_{l}^{P}|l=1,..,n\prime \right\}$ plotted against the index *l* after sorting them in descending order. As shown in Figure 1(a), the eigenvalues decrease drastically and most of the sum of the eigenvalues is concentrated in the first few eigenvalues. Therefore, in this paper, we use the projection vector $w_{1}^{P}={\left[w_{11}^{P},..,w_{1n}^{P}\right]}^{T}$, which corresponds to the largest eigenvalue, in order to remove input variables that are corrupted by noise. We first evaluate each input variable with the magnitude of $w_{1i}^{P}$. Then, with a threshold *M_w^p^_*, which is an average value of $\left|w_{1i}^{P}\right|$s, we obtain the modified data sample $x_{k}^{\prime }={\left[x_{k1}^{\prime },..,x_{kn}^{\prime }\right]}^{T}$ from the original data sample **x***_k_* = [*x_k_*_1_,.., *x_kn_*]*^T^* as follows:

(1) $$\{\begin{array}{ll} x_{\mathrm{ki}}^{\prime }=x_{\mathrm{ki}}, & \mathrm{if}||w_{1i}^{P}||\geq M_{wp}\\ x_{\mathrm{ki}}^{\prime }=0, & \mathrm{otherwise} \end{array}$$

### 3.2. Data Refinement by the Use of LDA Feature Feedback

In order to distinguish important parts for classification in a data sample, we feed discriminant features back to $x_{k}^{\prime }$. The discriminant features are extracted by using LDA. In the case of e-nose data, since the dimension of the data (*n*) is usually much larger than the number of available sample (*N*), the Small Sample Size (SSS) problem [21] occurs in extracting the features by using LDA. To resolve this problem, PCA is applied to *S_W_* to reduce the dimension of the input space to the rank of *S_W_*, and then LDA is applied to obtain *n*′(≪ *n*) projection vectors $w_{l}^{L},l=1,..,n\prime$ [18]. Figure 1(b) shows the eigenvalues $\left\{\lambda_{l}^{L}|l=1,..,n\prime \right\}$ plotted against the index *l* after sorting them in descending order. As shown in Figure 1(b), the sum of three largest eigenvalues, $\lambda_{l}^{L},l=1,2,3$ amounts to approximately 99% of the total sum of eigenvalues, so we use three projection vectors, $w_{l}^{L},l=1,2,3$, for the feature feedback procedure. Defining *α_l_* and *T* as the average value of $\left|w_{\mathrm{li}}^{L}\right|$ and a threshold, we can make a feature mask **m***_l_* = [*m_l_*_1_, *m_l_*_2_,*.., m_ln_*]*^T^*, *l* = 1, 2, 3, for each projection vector whose elements are either 1 or 0 as follows:

(2) $$\{\begin{array}{ll} m_{\mathrm{li}}=1, & \mathrm{if}|w_{1i}^{L}|\geq \alpha_{l}+T\\ m_{\mathrm{li}}=0, & \mathrm{otherwise} \end{array}$$

Finally, these three feature masks are merged by using OR operation as

(3) $$m=m_{1}⊕m_{2}⊕m_{3}$$

and the refined data sample **r***_k_* = [*r_k_*_1_,.., *r_kn_*]*^T^* is obtained by using the final feature mask **m** as defined by

(4) $$\{\begin{array}{ll} r_{\mathrm{ki}}=x_{\mathrm{ki}}, & \mathrm{if}\;m_{i}=1\\ r_{\mathrm{ki}}=0, & \mathrm{otherwise} \end{array}$$

The procedure of the overall data refinement can be summarized as follows:

**First stage - noise reduction**
- **Step 1** : Obtain the projection vector $w_{1}^{P}={\left[w_{1i},..,w_{1n}\right]}^{T}$ from the original data by using PCA.
- **Step 2** : On the basis of the average value of $w_{1i}^{P}$s, obtain the modified data $x_{k}^{\prime }$, where noise is reduced, by using (1).

**Second stage - data refinement**
- **Step 3** : With the modified data $x_{k}^{\prime }$, produce projection vectors $w_{l}^{L},l=1,2,3$, by using LDA.
- **Step 4** : For each projection vector obtained at Step 3, produce three feature masks **m***_l_*, *l* = 1, 2, 3, by using (2).
- **Step 5** : Merge three feature masks to mask the final mask **m** as follows:
  $$m=m_{1}⊕m_{2}⊕m_{3}$$
- **Step 6** : Obtain the refined data **r***_k_* by multiplying the final mask **m** to **x***_k_* element by element.

**Figure 2.**
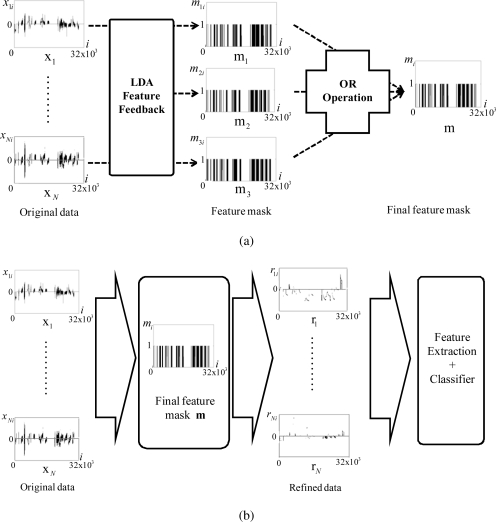
The procedure of the overall data refinement and vapor classification. **(a)** Feature feedback to obtain the final feature mask; **(b)** Vapor classification based on the data refinement.

The procedure of the proposed data refinement and vapor classification is shown in Figure 2.

## 3.3. Example

**Figure 3.**
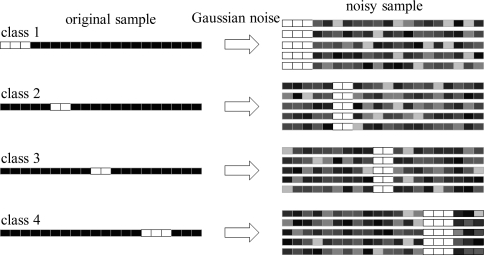
The vector sample from four classes (the left samples: without noise, the right samples: with Gaussian noise).

To show the effectiveness of the proposed data refining method, a toy example is presented. Let us consider a set of 20 vector samples (∈ ℝ^20^) whose input variables are either 255 or 0. In Figure 3, these variables are represented as white and black pixels, respectively. Each sample belongs to one of four classes, and its class can be identified by the position of the white pixels. The number of white pixels is 3, 2, 2 or 3 depending on the class. We add Gaussian random noise with standard deviation of 15 to each sample so that the peak signal to noise ratio (PSNR) of a sample is 24 ∼ 29 dB (see Figure 3). It is obvious that the input variables corresponding to the white pixels (total 10 variables) have the most discriminative information because their variances in the same classes are zero, while those in the different classes are very large. We apply the proposed data refining method to this data set and observe which variables were first removed as the number of selected pixels (*n*_s_) decreases from 20 to 10. As shown in Table 2, when removing the input variables by using the proposed method, only the noisy variables corresponding to black pixels are first eliminated, while the input variables corresponding to white pixels remain. From this, we can see that the proposed data refining method is very effective in removing the input variables that have little discriminative power. Removing noisy input variables can be helpful in extracting better discriminant features. This can be observed by investigating PSNR in a sample after the variable selection. Given an original sample **x***_o_* = [*x_o_*_1_, *x_o_*_2_,.., *x_on_*__s__]*^T^* and a noisy sample **x***_N_* = [*x_N_*_1_, *x_N_*_2_,.., *x_Nn_*__s__]*^T^*, the PSNR of sample **x***_N_* is computed as

(5) $$\text{PSNR}=20\cdot \log_{10}(\frac{{\mathrm{MAX}}_{x_{o}}}{\sqrt{\mathrm{MSE}}})$$

where

(6) $$\mathrm{MSE}=\frac{1}{n_{s}}\sum_{i=1}^{n_{s}}{\left\|x_{oi}-x_{Ni}\right\|}^{2}$$

**Figure 4.**
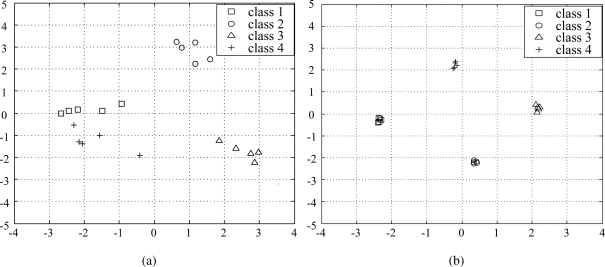
Sample distribution for various *n_s_* in two principal component axes. **(a)** original data sample (*n_s_* = 20); **(b)** refined data sample (*n_s_* = 10).

Here, *MAX*_**x**_*o*__ is the maximum value of an input variable. As can be seen in Table 2, the average PSNR of the samples after removing noisy input variables, increases as *n*_s_ decreases. Therefore, by properly setting a threshold *T*, we can obtain samples that contain most of the discriminative information and, at the same time, a higher PSNR. In order to see how the data samples are distributed before and after applying the proposed data refining method, we plot the samples with Gaussian noise (the samples in the right side of Figure 3) and the samples after applying the proposed method for *n_s_* = 10 in the subspace consisting of two principal axes. As shown in Figure 4(a), while the samples belonging to the same class are widely scattered, they become more closely clustered after removing the noisy input variables (Figure 4(b)).

## 4. Channel Selection for Vapor Classification

The gas sensor array chip used in our e-nose system consists of 16 separate channels with an interdigitated electrode, microheater, and micromachined membrane for further temperature-controlled measurement applications. We redesigned the e-nose system to make it more effective in computational time and classification performance. The redesigned sensor array consists of some channels instead of using all 16 channels. The usefulness of each channel is evaluated based on the feature mask obtained in Section 3. Each data sample is measured through 16 channel over 2,000 time points and can be represented as not only a 16 × 2, 000 matrix (**X***_k_*) but also a 32000-dimensional vector **x***_k_* ∈ ℝ^32000^ by use of the lexicographic ordering operator. As with the data *X_k_* in matrix form, the final mask **m** can be also represented as a matrix of 16 × 2, 000 (**M**). From the distribution of elements of **M**, we can see which channels play an important role for vapor classification, *i.e.*, the *i*-th channel containing more elements (*M_i,j_*, *i* = 1,.., 16, *j* = 1,.., 2000,) that equal 1 is considered as more important channel. Such important channels are selected for the redesigned sensor arrays, while less important channels are discarded. The procedure of the proposed channel selection can be summarized as follows:

- **Step 1** : Produce projection vectors $w_{l}^{L},l=1,2,3$, by using LDA.
- **Step 2** : Obtain the feature mask **m** by using these projection vectors as in Subsection 3.2, and transform **m** into a matrix form **M**.
- **Step 3** : For each channel, count the number of elements (*M_i,j_*) that equal 1.
- **Step 4** : Select channels with higher counts.

## 5. Experimental Results

**Figure 5.**
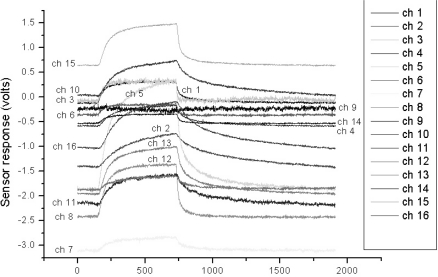
Typical time-responses of 16 channel sensor array with respect to inflow of acetone vapor at 5,000 ppm.

**Figure 6.**
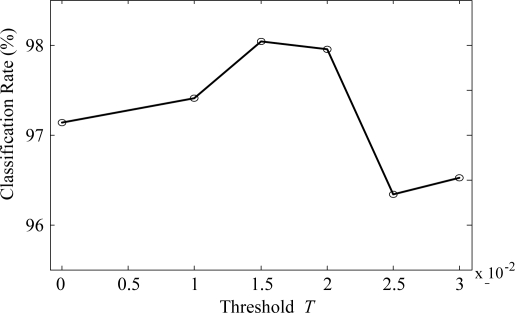
Classification rates for various threshold *T*.

In order to evaluate and compare the performance of the proposed algorithm, we utilized the VOC measurement data set as in Section 3. Figure 5 shows one of the typical multi-sensor responses of the acetone vapor. We checked the classification rates by increasing *T* from 0 to 0.02 in order to find a suitable threshold *T*. As can be seen in Figure 6, the classification rate does not always increase as *T* increases, *i.e.*, the classification rate rises as *T* increases up to 0.015, and then drops with further increase in *T*. From these results, we set the threshold values to 0.015 for all the experiments.

### 5.1. Data Refinement for Vapor Classification

**Figure 7.**
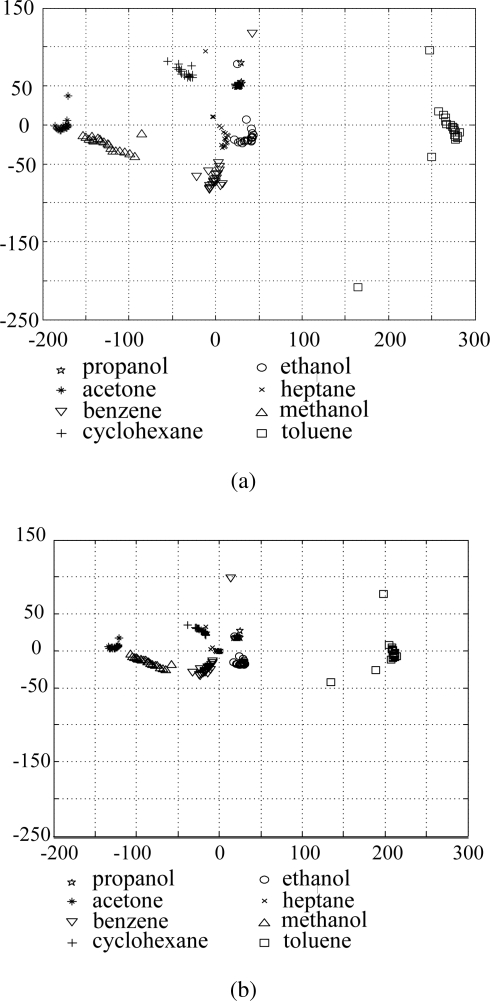
Distribution of the original data and refined data in PCA feature space. **(a)** Original data; **(b)** Refined data.

In order to compare the results before and after applying the proposed data refining method, we plot the original data and the refined data in the subspace consisted of two principal axes, which is shown in Figure 7(a) and 7(b), respectively. For the original data in Figure 7(a), samples belonging to the same class are widely scattered, and some samples of acetone and benzene are overlapped with each other. On the contrary, in the refined data of Figure 7(b), the samples of the same class are clustered more closely and there is less overlap between samples belonging to different classes.

**Figure 8.**
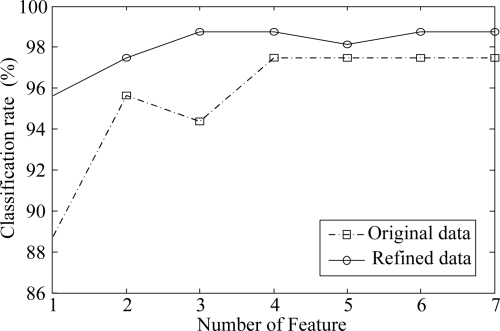
Classification rates for different number of features.

In order to evaluate the classification rates, we perform 10-fold cross validation [22] 10 times and computed the average classification rate. In this scheme, one sample from each class was randomly selected for testing, while the remaining samples were used for training. In other words, there were 152 data samples in the training set and 8 images for testing. Each input variable of data samples in the training set was also normalized using the mean, and variance of the training set. The features for classification are extracted by LDA, which is a well known method for dealing with classification problems. One nearest neighbor rule was used as a classifier and the *l*_2_ norm was used to measure the distance between two samples. Figure 8 shows the classification rates for different numbers of features. As can be seen in Figure 8, the refined data gives about 4.0% higher recognition rates on average over all the number of features than does the original data. From the Figures 7 and 8, we can see that the proposed method effectively refines the data by removing unnecessary input variables, which make it suitable for vapor classification.

### 5.2. Channel Selection for Vapor Classification

In the feature mask in a form of matrix of 16×2000, **M**, each row and column represents each channel and each time point, respectively. The elements of **M**, {*M_i,j_* |*i* = 1,.., 16, *j* = 1,.., 2000}, are either 1 or 0. Table 3 shows the number of *M_i,j_*s that equal to 1 for each channel when *T* = 0.015. As mentioned in Section 4, the input variables corresponding to *M_i,j_* = 1 contain the most useful information for classification. Therefore, the channels containing the largest number of *M_i,j_*s that are equal to 1 are first selected, in order to construct a new sensor array. Table 4 shows the classification rates for different numbers of features as the number of channels of the sensor array is increased. As can be seen in Table 4, we can obtain the best classification rate when designing the sensor array with only 9 top channels, which is 2.3% higher than is achieved by the use of all the channels. This result means that some channels with a lower rank may interfere somewhat with the acquisition of useful information for classification. So those channels are removed by the use of the proposed channel selection method.

## 6. Conclusions

In this paper, we proposed a new data refinement and channel selection method for vapor classification in the portable e-nose system. In the real environment, the data measured by a portable e-nose system is likely to be corrupted by noise, which interferes with feature extraction for classification. For an e-nose system that is robust in various environments, we use the features, which are extracted by PCA, to reduce the noise in the originally measured data. Then, by using the features extracted by LDA, we distinguished the important part with contains more discriminative information in the data. Through the example in Subsection 3.3, we showed that the proposed data refining method can increase the PSNR of the refined data and also maintain most of the discriminative information. An improvement in classification rates is then to be expected because the redundant and unnecessary information, which can be the noise in the classification, is discarded. The proposed data refining method allows significant computational saving depending on the size of the refined data. Reducing the computational complexity and data sample size have become more important when many applications are used in various mobile devices including a portable e-nose.

We also designed a new sensor array in order to obtain for a more efficient e-nose system involving only some channels, which turn out to be the most significant, instead of using all the channels. The channels are selected on the basis of the feature mask that is used in the data refinement. Using only the useful channels, both the classification performance and the efficiency of the e-nose system in computation and data storage are improved. In the proposed method, we require a more systematic algorithm in order to decide the optimal *T* and the number of the selected channels. These objectives remain as future work.

## Figures and Tables

**Table 1.** Characteristics of PCA and LDA.

Method	Scatter matrix used	Objective function
PCA	$S_{T}=\sum_{i=1}^{N}\left(x_{i}-\mu \right){\left(x_{i}-\mu \right)}^{T}$	*W^P^* arg max*_w_* |*W^T^S_T_W*|
LDA	$\begin{array}{c} S_{B}=\sum_{i=1}^{c}N_{i}\left(\mu_{i}-\mu \right){\left(\mu_{i}-\mu \right)}^{T}\\ S_{W}=\sum_{i=1}^{c}\sum_{x_{k}\in c_{i}}\left(x_{k}-\mu_{i}\right){\left(x_{k}-\mu_{i}\right)}^{T} \end{array}$	$W^{L}\text{arg}\;{\text{max}}_{W}\frac{\left|W^{T}S_{B}W\right|}{\left|W^{T}S_{W}W\right|}$

*μ* : mean of the whole training samples.

*μ_i_* : mean of the samples belonging to class *c_i_* that has *N_i_* samples.

**Table 2.** Number of input variables corresponding to white and black pixels remained for various values of *n_s_*.

*n_s_*	20	18	16	14	12
No. of white pixels remained	10	10	10	10	10
No. of black pixels remained	10	8	6	4	2
PSNR *	25.3	25.5	25.5	25.7	26.0

*: average PSNR of the samples that consist of ns input variables.

**Table 3.** The number of *M_i,j_*s that equal to 1 for each channel in the feature mask **M**.

Channel index	1	2	3	4	5	6	7	8
No. elements of one	1967	1728	1104	534	1053	887	238	1944
Channel index	9	10	11	12	13	14	15	16
No. elements of one	791	644	284	30	346	1284	653	994

**Table 4.** Classification rates for different number of features as increasing the number of channels of the sensor array.

Feature	1	2	3	4	5	6	7	aver.
Channel index								
1,8,2,14,3	87.5	96.9	96.9	96.9	96.9	96.9	96.9	95.5
1,8,2,14,3,5	84.4	97.5	96.3	97.5	96.9	98.1	98.1	95.5
1,8,2,14,3,5,16	79.4	96.3	97.5	98.1	98.1	98.1	98.1	95.1
1,8,2,14,3,5,16,6	95.0	96.3	96.9	98.1	97.5	97.5	97.5	97.0
1,8,2,14,3,5,16,6,9	93.1	98.1	98.1	98.8	98.8	98.8	98.8	97.8
1,8,2,14,3,5,16,6,9,15	86.3	96.9	96.2	97.5	97.5	97.5	98.1	95.7
1,8,2,14,3,5,16,6,9,15,10	86.9	96.9	96.9	97.5	97.5	97.5	97.5	95.8
all channels	88.8	95.6	94.4	97.5	97.5	97.5	97.5	95.5
